# Establishing Porcine Monocyte-Derived Macrophage and Dendritic Cell Systems for Studying the Interaction with PRRSV-1

**DOI:** 10.3389/fmicb.2016.00832

**Published:** 2016-06-02

**Authors:** Helen Singleton, Simon P. Graham, Katherine B. Bodman-Smith, Jean-Pierre Frossard, Falko Steinbach

**Affiliations:** ^1^Virology Department, Animal and Plant Health AgencySurrey, UK; ^2^Faculty of Health and Medical Sciences, University of SurreySurrey, UK

**Keywords:** PRRSV, macrophage, dendritic cell, CD163, CD169

## Abstract

Monocyte-derived macrophages (MoMØ) and monocyte-derived dendritic cells (MoDC) are two model systems well established in human and rodent systems that can be used to study the interaction of pathogens with host cells. Porcine reproductive and respiratory syndrome virus (PRRSV) is known to infect myeloid cells, such as macrophages (MØ) and dendritic cells (DC). Therefore, this study aimed to establish systems for the differentiation and characterization of MoMØ and MoDC for subsequent infection with PRRSV-1. M-CSF differentiated MoMØ were stimulated with activators for classical (M1) or alternative (M2) activation. GM-CSF and IL-4 generated MoDC were activated with the well established maturation cocktail containing PAMPs and cytokines. In addition, MoMØ and MoDC were treated with dexamethasone and IL-10, which are known immuno-suppressive reagents. Cells were characterized by morphology, phenotype, and function and porcine MØ subsets highlighted some divergence from described human counterparts, while MoDC, appeared more similar to mouse and human DCs. The infection with PRRSV-1 strain Lena demonstrated different replication kinetics between MoMØ and MoDC and within subsets of each cell type. While MoMØ susceptibility was significantly increased by dexamethasone and IL-10 with an accompanying increase in CD163/CD169 expression, MoDC supported only a minimal replication of PRRSV These findings underline the high variability in the susceptibility of porcine myeloid cells toward PRRSV-1 infection.

## Introduction

Myeloid cells differentiate into various types of mononuclear phagocytic cells among them MØs and DCs. In order to facilitate a range of complex functions, both MØs and DCs demonstrate phenotypic and functional heterogeneity according to their activation or maturation state. It is the pathogen/disease situation and signals from the surrounding microenvironment, which determine the state of MØs and DCs, resulting in several subsets that in turn determine the disease outcome. The study and characterization of myeloid cells is, therefore, an essential starting point in understanding virus kinetics and interactions with host cells. Current understanding of myeloid cells is based on studies using human cells or mouse models, whereas other species are not characterized to the same extent. Since important differences have been highlighted between mouse and human myeloid cell systems, the understanding of myeloid cells is equally important to aid the understanding of veterinary diseases.

Activated MØs contribute to specific functional roles within the immune response (Gordon and Taylor, 2005). Two MØ subsets are recognized, referred to as M1 and M2, which result from classical or alternative activation, respectively (Nathan, 1991; Gordon, 2003). Classical (M1) activation of MØ requires two signals, namely IFN-γ and TLR ligation (Mosser, 2003), and can be generated *in vitro* using IFN-γ and LPS (Nathan, 1991; Held et al., 1999). M1 macrophages are able to kill intracellular pathogens (Mosser and Edwards, 2008), and pro-inflammatory cytokines including IL-1β, TNF, IL-6, IL-12, and IL-23 (Verreck et al., 2004; Mantovani et al., 2005). In response to LPS, mouse M1 produce inducible nitric oxide synthase (iNOS; MacMicking et al., 1997), whereas human macrophages do not (Thoma-Uszynski et al., 2001).

Alternative (M2) activation of macrophages occurs via IL-4 or IL-13 (Stein et al., 1992). Resulting macrophages show increased mannose receptor expression (CD206) and are distinct from M1 MØs by their limited killing ability (Modolell et al., 1995). M2 MØs are associated with wound repair (Gordon, 2003), producing components for extracellular matrix synthesis (Gratchev et al., 2001). Other alternative activation of macrophages occurs with IL-10, glucocorticoids, and vitamin D_3_. Although the ‘M2’ nomenclature is often also applied to these cells, they show little similarity with IL-4/IL-13 M2 activated MØs (Mantovani et al., 2004).

Myeloid DCs also exist as different subsets according to their activation. In tissues, DCs reside in an immature state, unable to stimulate T-cells. iDCs are well equipped for antigen uptake via phagocytosis (Svensson et al., 1997), macropinocytosis (Sallusto et al., 1995), or receptor-mediated endocytosis (Sallusto and Lanzavecchia, 1994; Jiang et al., 1995), but maturation of DCs and accessory signals (e.g., CD80/86) required for T-cell activation are necessary for primary immune responses. DC maturation occurs by way of ‘danger signals.’ This can be mimicked *in vitro* using a cocktail of factors including TLR ligands, such as LPS, inflammatory cytokines (TNF-α, IL1-β, and IL-6), and molecules released following tissue damage such as PGE_2_ (Scandella et al., 2002; Jeras et al., 2005).

Significant differences have also been identified between mouse and human DC subtypes (Vereyken et al., 2011). Comparative analysis suggests that the pig’s immune system is more closely resembled to that of the human (Schook et al., 2005), but pigs are important in their own right as the most important meat producing mammalian livestock species worldwide, and host to several pathogens, including zoonoses.

An important disease of swine is PRRS, caused by the virus PRRSV, which infects cells of myeloid lineage (Snijder and Meulenberg, 1998), the proposed targets being alveolar macrophages and other tissue macrophages, but less so monocytes and DCs (Haynes et al., 1997; Van Gorp et al., 2008). PRRSV, belonging to genus *Arterivirus* (Snijder and Meulenberg, 1998; Meulenberg, 2000) is responsible for respiratory disease in pigs and reproductive failure in sows, affecting the swine industry worldwide (Hopper et al., 1992; Done and Paton, 1995; Rossow, 1998). Having emerged in North America during the late 1980s, PRRSV was identified in Europe shortly afterward (Lindhaus and Lindhaus, 1991). PRRSV-1 (European) and PRRSV-2 (North American), cause a similar syndrome, despite sharing only 55–70% nucleotide identity (Forsberg et al., 2002), which has led to the suggestion to consider these as separate virus species. Sequence analysis of PRRSV-1 strains defined at least three distinct subtypes, namely subtype 1 (pan-European) and Eastern European subtypes 2 and 3 (Stadejek et al., 2008, 2013). PRRSV isolates show significant differences in virulence and highly pathogenic (HP) PRRSV strains first arose in PRRSV-2 strains (Tong et al., 2007), but were since also identified in PRRSV-1 subtype 3 such as strains Lena and SU1-Bel (Karniychuk et al., 2010; Morgan et al., 2013; Weesendorp et al., 2013).

Porcine reproductive and respiratory syndrome virus has a restricted cell tropism and infection of porcine alveolar macrophages is well described *in vitro* and *in vivo* (Haynes et al., 1997; Gomez-Laguna et al., 2013), although variability in macrophage susceptibility was observed *in vitro* (Duan et al., 1997a; Vincent et al., 2005) and peritoneal macrophages as well as macrophage precursor cells, i.e., bone marrow cells and peripheral blood monocytes, are reportedly refractory to PRRSV infection (Duan et al., 1997a,b; Teifke et al., 2001). PRRSV has been detected in or isolated from macrophages of various tissues, including the spleen, liver, Peyer’s patches, thymus, and placenta (Larochelle et al., 1996; Sur et al., 1996; Duan et al., 1997a,b; Lawson et al., 1997; Karniychuk and Nauwynck, 2009). In contrast, PRRSV infection of DCs is poorly understood and there are possibly significant differences between PRRSV-1 and -2. PRRSV-2 infection of MoDC is frequently described (Wang et al., 2007; Flores-Mendoza et al., 2008; Park et al., 2008) and infection of bone marrow derived DCs (BMDC) was apparent (Chang et al., 2008), whereas reports of PRRSV-1 infection of DCs are very few (Silva-Campa et al., 2010).

It was hypothesized that PRRSV is able to elicit immunosuppression (Drew, 2000; Diaz et al., 2005), although no direct evidence of such by PRRSV-1 exists to date (Mateu and Diaz, 2008). More detailed reviews of host interactions with PRRSV-1 conclude that most PRRSV-1 strains initiate weak innate immune responses, resulting in prolonged viremia and persistent infection, whereas strains that induce a significant inflammation are cleared more effectively (Morgan et al., 2013; Weesendorp et al., 2013; Salguero et al., 2015). However, previous *in vitro* studies of PRRSV-2 imply that it impairs DC function directly by modulation of important molecules, including the down-regulation of MHC-I and MHC-II (Loving et al., 2007; Wang et al., 2007; Park et al., 2008). This suggested PRRSV-2 infected DCs were less efficient at presenting antigens to T cells.

Although well described in humans and mice, differentiation of monocytes to MØs *in vitro* is not well established for pigs, although studies using L929-conditioned media as a source of M-CSF indicate its feasibility (Mayer, 1983; Genovesi et al., 1990) and human M-CSF has been used to generate porcine macrophages from bone marrow (Kapetanovic et al., 2012), which expressed macrophage markers (CD14, CD16, and CD172a), and were phagocytic. Indicative of classical activation, these responded to LPS treatment by TNF-α production, but like human M1 MØs, lack NO production (Kapetanovic et al., 2012). MoMØ showed an altered phenotype compared to monocytes, including the expression of porcine macrophage marker CD203a (McCullough et al., 1997, 1999). Few studies of porcine M1 and M2 phenotypes generated from MoMØ have yet been carried out, and it is important to further characterize porcine macrophages (Ezquerra et al., 2009).

*In vitro* generation of DCs from monocytes (MoDC) using growth factor GM-CSF and IL-4 is established in various species, including cats (Mizukoshi et al., 2009), horses (Moyo et al., 2013), and cattle (Howard et al., 1999). Porcine MoDC generation from was reported before, using slightly different conditions (Carrasco et al., 2001; Paillot et al., 2001).

Porcine reproductive and respiratory syndrome virus 1 entry is thought to occur *via* receptor-mediated endocytosis. CD163 and sialoadhesin (CD169) were considered essential for PRRSV-1 entry in macrophages (Van Breedam et al., 2010a). CD169, a type 1 transmembrane protein restricted to macrophages (Munday et al., 1999), directly binds to sialic acids present on M/GP5 glycoprotein complexes in the PRRSV envelope. Transfection of CD169 into non-permissive cell lines enabled PRRSV attachment and internalization *via* endocytosis (Vanderheijden et al., 2003; Van Breedam et al., 2010b), but not productive infection, suggesting that an additional factor was required. CD163, also a type 1 transmembrane glycoprotein expressed mainly on certain monocytes and macrophages (Hogger et al., 1998), is implicated in later stages of PRRSV entry (Van Breedam et al., 2010a), considered essential for genome release, potentially requiring interaction with the minor envelope glycoproteins GP2a and GP4 (Das et al., 2010).

As investigations of MoMØ and MoDC subsets in pigs remain elusive, our aim was to describe both cell types *in vitro*, distinguishing different sub-populations by phenotypical and functional analysis, and using them to assess how these cells react to PRRSV-1 infection with a highly pathogenic strain (Lena).

## Materials and Methods

### Porcine Myeloid Cell Isolation and Culture

All porcine primary cells were collected from Large White cross Landrace pigs under the age of 2 years. All work was carried out under license from the UK Home Office (PPL 70/7057) under the Animal Act 1986 and approved by the ethics committee at APHA. Briefly, approximately 200 ml of venous blood was collected into sterile duran bottles containing 25 IU of heparin sodium (LEO, Ballerup, Denmark) to prevent blood coagulation. Each 30 ml was layered onto 20 ml Biocoll separating solution, 1.077 g/ml density (Biochrom, Berlin, Germany) and centrifuged at 1455 × *g* for 30 min at room temperature. The PBMC interface was removed and washed with 4°C Dulbecco’s PBS (PBS; Invitrogen, Paisley, UK). PBMC were counted and resuspended in 10 μl anti-human CD14 MicroBeads (Miltenyi Biotec, Gergisch Gladbach, Germany) per 10^7^ cells and incubated at room temperature for 12 min. After washing with PBS + 2% fetal bovine serum (FBS), cells were resuspended in 500 μl PBS + 2% FBS + 5 mM EDTA (Sigma, Poole, UK; MACS buffer) per 10^8^ cells and applied to a MACS LS column placed on a magnetic quadro MACS unit (Miltenyi Biotec). Flow through was collected as the CD14^-^ fraction and after washing the column with MACS buffer, the CD14^+^ fraction was collected in RPMI-1640 media +10% FBS, 100 IU/ml of penicillin, 100 μg/ml of streptomycin, and 50 μg/ml of gentamicin (all Invitrogen; complete tissue culture [TC] medium) and cultured on ultra-low bind (ULB) plates at 37°C with 5% CO_2._

For differentiation of monocytes to MoMØ, freshly isolated monocytes were cultured at a cell density of 1 × 10^6^/ml in complete TC medium supplemented with 50 ng/ml of recombinant human M-CSF (Miltenyi Biotec) for 4 days. For differentiation of monocytes to MoDC, freshly isolated monocytes were cultured at a cell density of 2 × 10^6^/ml (1 ml/well) in complete TC medium supplemented with 10 ng/ml of recombinant porcine GM-CSF and 10 ng/ml of recombinant porcine IL-4 (R&D Systems, Abingdon, UK) for 4 days. Cell differentiation was monitored by assessment of cell morphology using light microscopy and phenotypic and functional characterization.

For MoMØ activation, culture medium was replaced after 4 days with fresh TC medium containing 10 ng/ml of LPS (from *Salmonella Minnesota*; Enzo Life Sciences, Exeter, UK) and 100 ng/ml of recombinant porcine IFN-γ (R&D Systems) for classical activation of MoMØ (M1 macrophages). For alternative activation (M2 macrophages), 10 ng/ml of recombinant porcine IL-4 was added. Alternatively MoMØ were also treated with 10 μg/ml of water soluble dexamethasone (Sigma) or 10 ng/ml of recombinant porcine IL-10 (R&D Systems) for 24 h.

Monocyte-derived dendritic cells were treated with a maturation cocktail for 24 h. This contained 100 ng/ml of LPS (*Salmonella Minnesota*), 100 ng/ml of porcine IFN-γ, 20 ng/ml of porcine TNF-α, 20 ng/ml of equine IL-6, 10 ng/ml of equine IL-1β, and 1 μg/ml of PGE_2_ (all R&D Systems).

### Functional Assays

Endocytosis was assessed using allophycocyanin (APC)-labeled ovalbumin (OVA; Invitrogen). Cells were resuspended in cold TC medium and added to 96-well round bottom plates at 1 × 10^5^/well. APC-OVA was added to cells at 20 μg/ml and incubated for 1 h at either 4°C (control) or 37°C. Cells without beads were used as a further negative control. Cells were washed three times with cold PBS, and stained for viability using LIVE/DEAD violet fixable dye (Invitrogen) before flow cytometric analysis.

Phagocytosis was assessed using fluorescein isothiocyanate (FITC)-labeled sulfate FluoSpheres^®^microsphere particles (4 μm diameter) or FITC-labeled carboxylate-modified microspheres (1 μm diameter; both Invitrogen). Cells were split between two wells of a 24-well ULB plate to allow for a control well without particles, and supplemented with 500 μl of fresh RPMI resulting in a cell density of 5 × 10^5^/ml. Microspheres were added at 2 × 10^5^ beads/ml and incubated for 3 h at either 4°C (control) or 37°C. Cells were then harvested and washed three times in cold PBS and subsequently stained for viability using LIVE/DEAD violet fixable dye before flow cytometric analysis.

### Flow Cytometry

All flow cytometry was carried out using a MACSQuant Analyzer flow cytometer (Miltenyi Biotec). Antibodies were added to cell pellets following harvesting from plates and centrifugation. For elimination of dead cells, cells were resuspended in LIVE/DEAD violet fixable dye and incubated at RT for 20 min protected from light.

For surface molecule staining, cells were added to round bottom 96-well plates (∼5 × 10^5^/well) and stained with relevant antibodies (**Table 1**) at 4°C for 30 min before washing in PBS + 1% FBS + 0.09% sodium azide (FACS buffer). Single color stained cells and unstained cells were used to calculate compensation for fluorescence spill-over. Individual samples were also stained with isotype negative controls of corresponding concentration to the relative antibody where described. Prior to analysis, cells were resuspended in CellFix (BD Biosciences, Oxford, UK).

**Table 1:** Table of antibodies.

Antibody	Host Species	Target Species	Clone	Isotype	Conjugate	Supplier
**Primary antibodies**						
SLA Class II DR	Mouse	Pig	2E9/13	IgG1	FITC	AbD Serotec
CD14	Mouse	Pig	MIL-2	IgG2b	FITC	AbD Serotec
CD163	Mouse	Pig	2A10/11	IgG1	FITC	AbD Serotec
CD169	Mouse	Pig	3B/11/11	IgG1	Unconjugated	AbD Serotec
CD152-muIg (CTLA4)	Mouse	Human		IgG2a	Unconjugated	Enzo Life Sciences
CD25	Mouse	Pig	K231.3B2	IgG1	Unconjugated	AbD Serotec
CD206 (α-MMR)	Goat	Human		Polyclonal	Biotinylated	R&D Systems
CD209 (DC-SIGN)	Sheep	Human		Polyclonal	Unconjugated	R&D Systems
CD83	Sheep	Human		Polyclonal	Biotinylated	R&D Systems
SWC9	Mouse	Pig	PM 18-7	IgG1	Unconjugated	Abcam
SDOW-17 Ascites	Mouse	Pig		IgG1	Unconjugated	Rural Technologies
**Secondary antibodies**						
G1-APC	Rat	Mouse	X56		APC	BD Biosciences
Donkey anti-goat IgG	Donkey	Goat			APC	Invitrogen
Donkey anti-sheep IgG	Donkey	Sheep			APC	Invitrogen
Sterptavidin-PE-Cy7					PE-Cy7	eBiosciences
**Alexa Fluor Zenon conjugates**						
IgG1 APC					APC	Life Technologies
IgG2a APC					APC	Life Technologies
IgG1 PE					PE	Life Technologies


For analysis of PRRSV infection cells were added to round bottom 96-well plates (∼5 × 10^5^/well) before being fixed and permeabilized using the CytoFix/CytoPerm kit according to the manufacturer’s protocol (BD Biosciences). PRRSV specific monoclonal antibody SDOW-17 (Rural Technologies Inc, Brookings, SD, USA) was diluted 1/20 in PermWash and 5 μl was added to each well and incubated for 30 min at 4°C. Anti-mouse IgG1 isotype control was used to assess for non-specific binding. Cells were washed twice, and stained with anti-mouse IgG1-APC conjugated secondary reagent (BD Biosciences).

### PRRSV-1 Virus Infection and Detection

Porcine reproductive and respiratory syndrome virus 1 strain Lena is a particularly pathogenic subtype 3 strain isolated from Belarus (Karniychuk et al., 2010) that was supplied by Prof. Hans Nauwynck (Ghent University, Ghent, Belgium). Virus was propagated and titrated on porcine alveolar macrophages prior to this study, as previously described (Morgan et al., 2013). Cells were infected with PRRSV-1 strain Lena at a multiplicity of infection (m.o.i.) of 0.1. Time-zero samples were obtained following 2 h of incubation with virus at 4°C to achieve attachment but no internalization of virus. At different time-points post infection (p.i.), cell supernatant was removed for analysis of infection by quantitative reverse transcriptase-polymerase chain reaction (qRT-PCR) and cells were harvested and centrifuged to obtain cell pellets. Cell pellets or supernatants were stored at -70°C prior to RNA extraction. A QiaAmp Viral RNA Mini Kit (Qiagen, Manchester, UK) was used to extract RNA from 140 μl of cell supernatant, or cells resuspended in 140 μl AVL buffer. RNA was eluted into 60 μl of elution buffer and stored at -20°C prior to RT-PCR analysis. For PRRSV detection by qRT-PCR, 2 μl of RNA was added to 23 μl of a PCR mastermix containing PRRSV-1 specific forward/reverse primers and probe (Frossard et al., 2012) and the Quantitect RT-PCR Kit (Qiagen). For RNA extracted from cells, a eukaryotic 18S rRNA RT-PCR was used as endogenous control to allow normalization (Applied Biosystems, Paisley, UK). qRT-PCRs were carried out using a Mx3000P real time PCR system (Agilent, La Jolla, CA, USA) as described before (Morgan et al., 2013).

To quantify PRRSV in cell supernatants, *C*_t_ values of PRRSV at 2 h p.i. (i.e., time-zero negative control) were subtracted from *C*_t_ values of PRRSV detected at each time-point p.i., providing a Δ*C*_t_, which was transformed into a fold increase as a measure of replication. Alternatively, where replication was measured in cells, relative quantitation was used to analyze changes between the time-zero negative control and time p.i., using normalization against 18S rRNA levels using the ΔΔ*C*_t_ method (Livak and Schmittgen, 2001).

### Statistical Analysis

Statistical tests were performed using GraphPad Prism Software, version 6.01. All experiments were performed independently at least three times using cells isolated from three different pigs unless stated otherwise. Statistical tests such as One-way or Two-way analysis of variance (ANOVA) and student *t*-tests were performed as detailed in the results.

## Results

### Differentiation and Characterization of Monocyte-Derived Macrophages (MoMØ)

**FIGURE 1:**
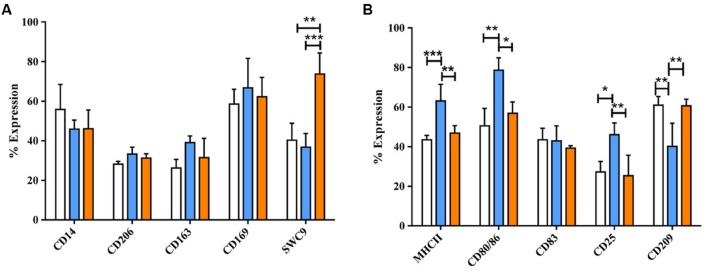
**Phenotypical analysis of porcine M1 and M2 MoMØs.** Freshly isolated peripheral porcine monocytes were treated with M-CSF for 4 days to obtain MoMØs. MoMØs were treated with IFN-γ and LPS to generate M1 macrophages (blue) or with IL-4 to generate M2 macrophages (orange), or MoMØs were left unstimulated (unfilled bars). After a further 24 h MoMØ were harvested and stained with various antibodies to assess their surface expression of pathogen recognition receptor/lineage markers **(A)** or antigen presentation/co-stimulatory molecules **(B)** for flow cytometry analysis. Data represent mean percentage of cells expressing markers +SD. One-way ANOVA was used to assess significance followed by Bonferroni’s multiple comparison test ^∗∗∗^*p* < 0.0001, ^∗∗^*p* < 0.001, ^∗^*p* < 0.05.

After 4 days with M-CSF, monocytes developed macrophage morphology (enlarged, adherent, round). Upon treatment for 24 h with LPS/IFN-γ (M1) these cells displayed increased formation of cell clusters, whereas IL-4 treated MoMØ (M2) had noticeably more elongated projections (**Supplementary Figure S1**). Surface expression of myeloid lineage and activation markers revealed that the percentage of M2 MoMØ expressing CD203a, was significantly higher than for unstimulated MoMØ (*p* < 0.001) and M1 MoMØ (*p* < 0.001), whereas expression of CD14, CD206, CD163, and CD169 remained unchanged (**Figure 1A**). However, MHC-II was detected on a significantly higher percentage of M1 MoMØ than on unstimulated MoMØ (*p* < 0.0001) and M2 MoMØ (*p* < 0.001) and the percentage of cells expressing CD80/86 was also significantly higher in M1 MoMØ, compared to unstimulated MoMØ (*p* < 0.001) and M2 MoMØ (*p* < 0.05). Further, more M1 MoMØ also expressed IL-2 receptor alpha CD25 (*p* < 0.05), whereas significantly less M1 MoMØ expressed CD209 (DC-SIGN) than unstimulated MoMØ (*p* < 0.001), and M2 MoMØ (*p* < 0.001; **Figure 1B**). CD83 expression was unchanged between unstimulated and M1 or M2 MoMØ. Endocytic and phagocytic activity of porcine monocyte-derived macrophages (PoMoMØ) following treatment with M1 or M2 activators was also assessed, but no significant differences were observed (**Supplementary Figures S2** and **S3**).

**FIGURE 2:**
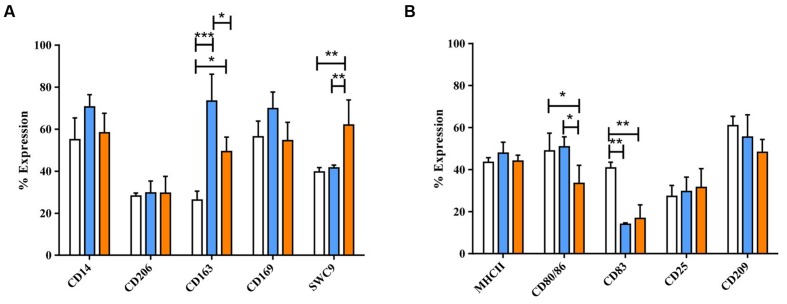
**Phenotypic analysis of dexa and IL-10 treated MoMØs.** Freshly isolated peripheral porcine monocytes were treated with M-CSF for 4 days to obtain MoMØs. MoMØs were treated with dexa (blue) or IL-10 (orange) or left unstimulated (unfilled bars). After a further 24 h MoMØs were harvested and surface stained for pathogen recognition receptors/lineage markers **(A)** or antigen presentation/co-stimulatory molecules **(B)** for flow cytometric analysis. Data represent mean percentage of cells expressing markers +SD. One-way ANOVA was used to assess significance followed by Bonferroni’s multiple comparison test ^∗∗∗^*p* < 0.0001, ^∗∗^*p* < 0.001, ^∗^*p* < 0.05.

Dexamethasone (dexa) and IL-10 were also applied to activate MoMØ. Light microscopy showed that dexa treated MoMØ appeared more rounded, with some enlarged cells compared to unstimulated MoMØ, while IL-10 treated MoMØ noticeably clustered together more frequently (**Supplementary Figure S1**). Thus both dexa and IL-10 treated MoMØ appeared unlike M1 and M2 MoMØ supporting the notion that they are not M2 macrophages (Gordon, 2003; Mantovani et al., 2005). Dexa and IL-10 treatment of MoMØ also resulted in two distinct MoMØ phenotypes, both showing differences to M1 and M2 MoMØ phenotypes. Dexa treated MoMØ showed significantly higher percentages of cells expressing CD163 (*p* < 0.0001), as did IL-10 treated MoMØ (*p* < 0.05), but the percentage of cells expressing CD163, was significantly higher in dexa MoMØ than IL-10 MoMØ (*p* < 0.005). IL-10 treated MoMØ showed significantly higher percentages of cells positive for CD203a than unstimulated (*p* < 0.001) and dexa treated MoMØ (*p* < 0.05). No differences were observed in the percentage expression of CD206 or CD169 in dexa or IL-10 treated MoMØ (**Figure 2A**). No differences were observed in the MHCII expression, but a lower proportion of IL-10 treated MoMØ expressed CD80/86 (*p* < 0.05). Both dexa and IL-10 treatment of MoMØ resulted in a decreased percentage of cells expressing CD83 (both *p* < 0.001), whereas no differences were observed in the percentage of cells positive for CD25 or CD209 (**Figure 2B**). Flow cytometric analysis determined that IL-10 treated MoMØ displayed significantly increased endocytosis (75.8%) compared with both dexa MoMØ (56.5%) and M2 MoMØ (57.2%; *p* < 0.05; **Supplementary Figure S4**). In two of five pigs, phagocytosing microsphere particles were also increased in dexa MoMØ (**Supplementary Figure S5**).

### PRRSV-1 Lena Infection of MoMØ Subsets

**FIGURE 3:**
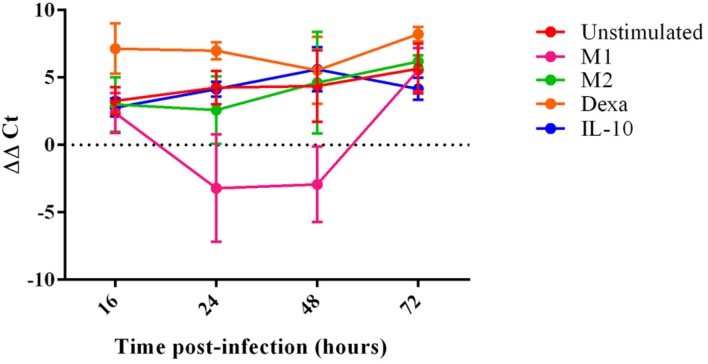
**PRRSV replication in MoMØs following different activation stimuli.** Monocytes were treated with M-CSF for 4 days to generate MoMØs, and either left unstimulated (red) or activated with LPS and IFN-γ (M1; pink), IL-4 (M2; green), dexa (orange) or IL-10 (blue) for 24 h before infection with PRRSV Lena using an m.o.i of 0.1. RNA was extracted from cells at either 16, 24, 48, or 72 h p.i, and a TaqMan qPCR was used to quantify PRRSV RNA. ΔΔ*C*_t_ represents difference between *C*_t_ at 2 h p.i (time zero) and *C*_t_ at each time point p.i after normalization against 18S RNA. Bars represent mean ΔΔ*C*_t_ ± SD.

**FIGURE 4:**
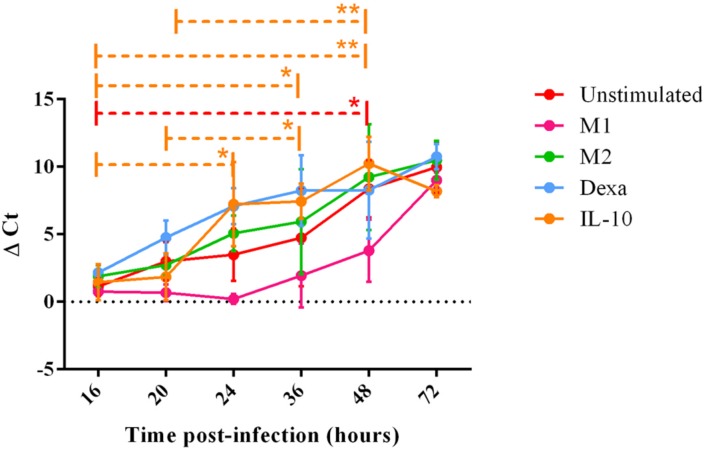
**PRRSV replication in MoMØ subset supernatant over time.** Monocytes were treated as in **Figure 3**. Viral RNA was extracted from cell supernatant at 16, 20, 24, 36, or 48 h p.i, and a TaqMan qPCR was used to obtain *C*_t_ values. Δ*C*_t_ represents difference between *C*_t_ at 2 h p.i (time zero) and *C*_t_ at each time point p.i shown at 16, 20, 24, 36, 48, or 72 h time-points in unstimulated (red), M1 (pink) M2 (green), dexa (blue) or IL-10 (orange) treated MoMØs. Lines represent mean Δ*C*_t_ ±SD in three independent experiments, each biological repeat tested in duplicate (*n* = 2 at 72 h p.i). ^∗∗^*p* < 0.001, ^∗^*p* < 0.05.

Porcine reproductive and respiratory syndrome virus 1 replication within MoMØ subsets was assessed by both qRT-PCR and flow cytometry at 16 h p.i. Only dexa treatment showed a significant increase in PRRSV-1 replication measurable by both methods (**Figure 3**, **Supplementary Figure S6**). In contrast, neither classical (M1) nor alternative (M2) macrophage activation resulted in changes at this time-point. After 16 h p.i., PRRSV-1 replication in dexa treated macrophages did not seem to increase further, and other MoMØ reached similar replication levels by around 72 h p.i. Interestingly, M1 MoMØ showed negative ΔΔ*C*_t_ values at 24 and 48 h (**Figure 3**), indicating a major obstacle for PRRSV-1 replication. However, PRRSV-RNA was detected in M1 cells after 72 h p.i. (**Figure 3**). In line with qRT-PCR results, only dexa MoMØ showed significant levels of PRRSV N protein expression (**Supplementary Figure S6**). At 16 h p.i., PRRSV-RNA levels in culture supernatants were low and no differences were observed between MoMØ subsets (**Figure 4**). At 20 h p.i., clear differences started to emerge, i.e., dexa MoMØ produced the highest amount of PRRSV-1, while M1 MoMØ did not show any significant PRRSV-1 production until around 48 h p.i.

### Characterization of Porcine MoDC

**FIGURE 5:**
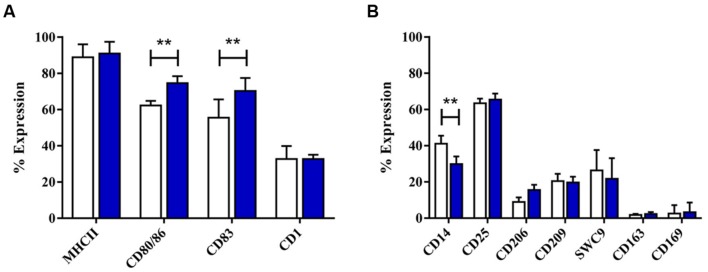
**The effect of maturation cocktail on porcine MoDC.** Four-day-old MoDC were treated with a maturation cocktail for 24 h before flow cytometric analysis of surface molecules involved in DC maturation or antigen presentation **(A)** and other co-stimulatory molecules and lineage markers **(B)** in untreated iMoDC (white bars) and cytokine maturation cocktail treated (blue bars) MoDCs. Bars show mean percentage of cells expressing each marker +SD. Two-way ANOVA was used to assess significance followed by Bonferroni’s multiple comparison test, ^∗∗^*p* < 0.001.

After 4 days with GM-CSF and IL-4, monocytes developed typical DC morphology, with cell clusters displaying surface protrusions. Twenty-four hours culture with the standard maturation cocktail resulted in no significant morphological changes, although maturation cocktail treated MoDC were less adherent than untreated MoDC (**Supplementary Figure S7**). DC maturation cocktail did induce some significant changes to MoDC phenotype, however. A significant increase was observed in expression of both CD80/86 (*p* < 0.001) and CD83 (*p* < 0.001), while MHC-II expression remained high (**Figure 5**). The number of maturation cocktail treated MoDC expressing CD14 was significantly lower than on immature (i) MoDC (*p* < 0.001); CD206 and CD209 (DC-SIGN) remained low, as did CD203a. The percentage of cells expressing CD163 and CD169 was negligible in both untreated and treated MoDC (**Figure 5B**).

**FIGURE 6:**
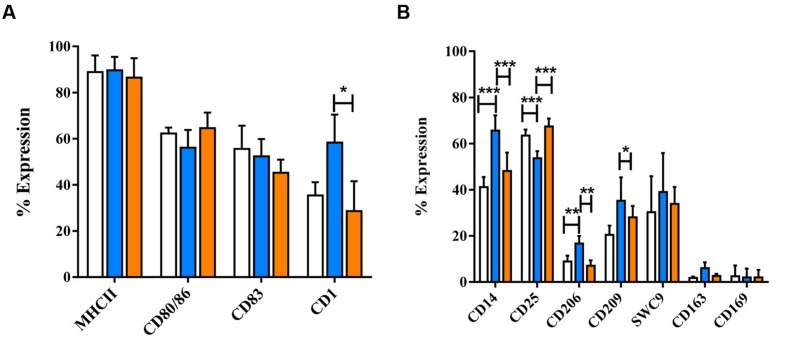
**The effects of dexamethasone and IL-10 treatment on the phenotype of porcine MoDCs.** Four-day-old MoDCs were treated with either dexamethasone or IL-10 for 24 h before surface staining with various anti-porcine or anti-human mono/poly-clonal antibodies for flow cytometric analysis of surface molecules involved in antigen presentation and maturation **(A)** or against other co-stimulatory molecules and myeloid lineage markers **(B)** in untreated MoDCs (unfilled bars), dexa treated MoDCs (blue bars), or IL-10 treated MoDCs (orange bars). Bars show mean percentage of cells expressing each marker +SD **(A,B)**. One-way ANOVA was used to assess significance followed by Bonferroni’s multiple comparison test ^∗∗∗^*p* < 0.0001, ^∗∗^*p* < 0.001, ^∗^*p* < 0.05.

Following 24 h treatment with dexa, MoDC appeared to have fewer and less dense clusters than untreated MoDC, but still maintained cellular elongation. In contrast, IL-10 treated MoDC appeared similar to untreated MoDC, with fewer cellular elongations (**Supplementary Figure S7**). Dexa treatment also resulted in a distinct MoDC phenotype, while IL-10 treatment rather maintained the iMoDC phenotype (**Figure 6**). Specifically the expression of CD1, CD14, CD206, and CD209 was up-regulated by dexa. Dexa MoDCs expressed almost twice as much CD14 and significantly more CD1 than maturation cocktail treated MoDC CD25 expression, however, was decreased following dexa treatment (*p* < 0.0001). In contrast to MoMØ, neither dexa nor IL-10 treatment affected expression of CD163 and CD169 on MoDC; the percentage of cells expressing these molecules remained below 10%.

**FIGURE 7:**
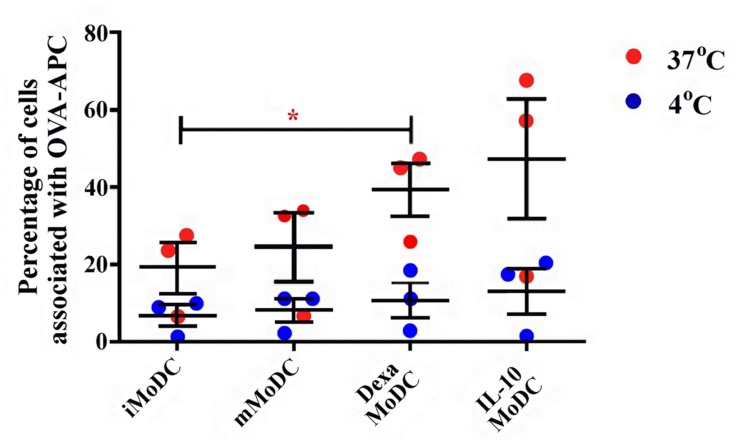
**The effect of dexa treatment on MoDC levels of endocytosis.** The ability of immature/iMoDC (untreated), mature/mMoDC (maturation cocktail treated) to endocytose APC^∗^-labeled OVA was assessed. Cells were incubated with OVA at 4°C as a negative control (blue dots), or at 37°C (red dots), for 1 h before flow cytometric analysis of APC^∗^ fluorescence. Each dot represents the percentage of cells fluorescing APC^∗^, indicating endocytic OVA uptake, in individual experiments. Lines represent mean percentages of cells associated with OVA-APC^∗^ ±SEM. One-way ANOVA was used to assess significance followed by Bonferroni’s multiple comparison test, ^∗^*p* < 0.05.

**FIGURE 8:**
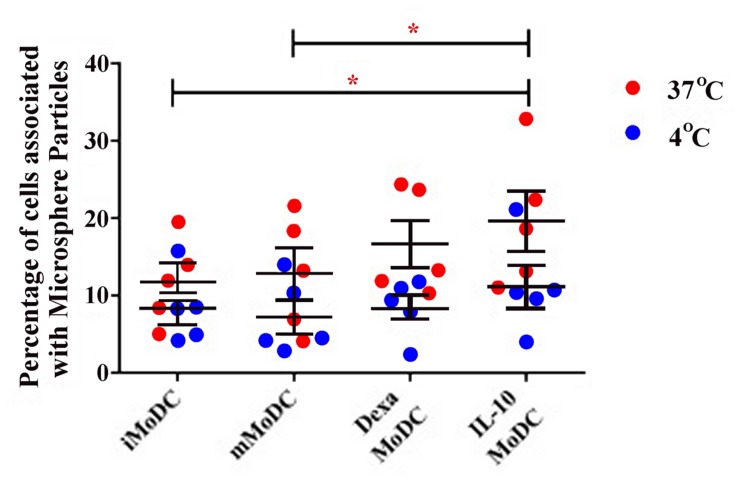
**The effect of IL-10 treatment on MoDC levels of phagocytosis.** The ability of untreated immature/iMoDC, maturation cocktail treated mature/mMoDC, dexa treated MoDC and IL-10 treated MoDC to phagocytose FITC-labeled microsphere particles was assessed. Cells were incubated with particles at 4°C as a negative control (blue dots), or at 37°C (red dots), for 3 h before flow cytometric analysis of FITC fluorescence. Each dot represents the percentage of cells fluorescing FITC, indicating phagocytic uptake, in individual experiments. Lines represent the mean percentage of cells associated with FITC microsphere particles ±SEM. ^∗^*p* < 0.05

Endocytosis was much lower in MoDC than observed in MoMØ. Percentages of cells associated with APC-OVA, indicative of endocytosis, were unchanged between iMoDC and mMoDC. In contrast, both dexa and IL-10 treatment of MoDC, appeared to increase endocytosis. However, replicate experiments were variable, and as a result only dexa treated MoDC showed a statistically significant increase in endocytic activity (*p* < 0.05; **Figure 7**). Levels of phagocytosis in MoDC subsets were also below those observed in MoMØ. Whilst maturation cocktail treatment did not induce changes, IL-10 treated MoDC showed statistically higher percentages of cells associated with FITC-labeled particles compared to iMoDC and mMoDC (*p* < 0.05; **Figure 8**).

### PRRSV Lena Infection of MoDC Subsets

**FIGURE 9:**
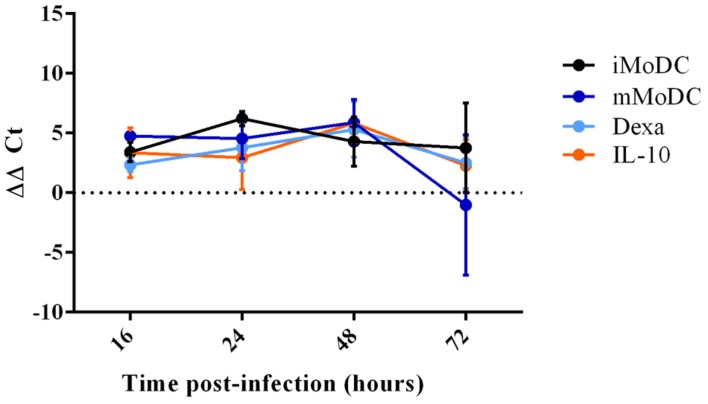
**qPCR detection of PRRSV replication in MoDC subsets at 16, 24, 48, and 72 h post infection.** Immature/iMoDC (black), maturation cocktail treated mature/mMoDC (dark blue), dexa treated MoDC (light blue), and IL-10 treated MoDC (orange), were infected with PRRSV Lena using an m.o.i of 0.1. Viral RNA was extracted from cells at either 16, 24, 48, or 72 h p.i and a TaqMan qPCR with an 18S endogenous control was used to obtain *C*_t_ values. ΔΔ*C*_t_ represents difference between *C*_t_ at 2 h p.i (time zero) and *C*_t_ at each time point p.i, both normalized to 18S. Bars represent mean ΔΔ*C*_t_ ± SD.

**FIGURE 10:**
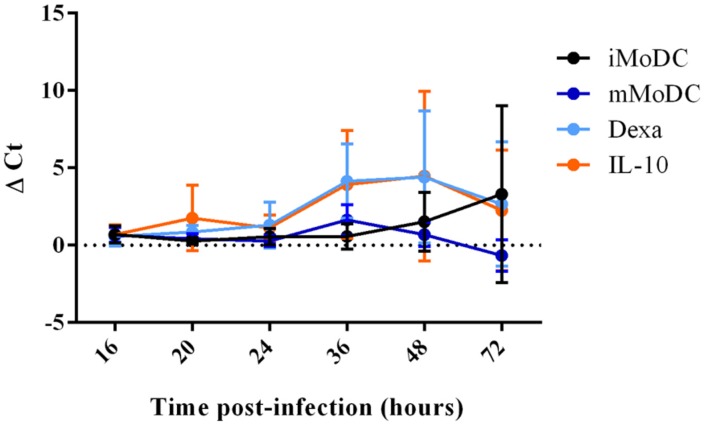
**PRRSV replication in MoDC supernatant between 16 and 72 h p.i.** Immature/iMoDC (black), maturation cocktail treated mature mMoDC (dark blue), dexa treated MoDC (light blue) and IL-10 treated MoDC (orange) for 24 h before infection with PRRSV Lena using an m.o.i of 0.1. Viral RNA was extracted from cell supernatant at 16, 20, 24, 36, 48, or 72 h p.i, and TaqMan qPCR was used to obtain *C*_t_ values. Δ*C*_t_ represents difference between *C*_t_ at 2 h p.i (time zero) and *C*_t_ at each time point p.i. Bars represent mean Δ*C*_t_ ± SD.

At 16 h p.i., viral replication was generally low in MoDC, with dexa MoDC being particularly inefficient and mMoDC displaying a slightly higher replication level. At 24 h p.i., viral replication appeared to increase but without showing significant differences between subsets (**Figure 9**). After 48 h viral replication in some MoDC subsets showed slight increases particularly dexa MoDC and IL-10 MoDC, but these differences were not significant. In line with qRT-PCR results, no PRRSV protein expression could be detected by intracellular flow cytometry staining at 20 h p.i. (not shown). At 16 h p.i., PRRSV was undetectable in MoDC supernatants, indicating a longer time for a single round of virus replication in all MoDC (**Figure 10**). Only after 36 h p.i. clear signs of viral production were seen in the supernatant of IL-10 and dexa treated MoDC, albeit at very low levels. This trend remained until the endpoint at 72 h p.i., with some evidence of virus production in iMoDC, whereas mMoDC seemed to be particularly refractory to PRRSV replication (**Figure 10**). Due to variability between biological repeats, no statistically significant differences were observed between MoDC subsets.

## Discussion

This study aimed to characterize subsets of macrophages and DCs derived from porcine monocytes, and to determine whether these cells could be used to explore PRRSV-1 infection kinetics within porcine myeloid cell sub-populations.

### Classical and Alternative Activation of Porcine MoMØ

Morphological and phenotypical analysis of poMoMØ treated with M1 (IFN-γ and LPS) or M2 cytokines (IL-4) resulted in two distinct populations, which are suggestive of different MØ activation pattern. Analysis of markers associated with classical and alternative activated macrophages in humans and mice suggested that despite some similarities, poMoMØ may not behave alike. Morphological changes were unexpected to be prominent, since human M1 and M2 macrophages lack any particular morphology (Porcheray et al., 2005; Vereyken et al., 2011). Our results align with this, as poMoMØ formed clusters suggestive of decreased adherence, consistent with Rey-Giraud et al. (2012) who describe increased detachment of human M1 macrophages. IL-4 treated poMoMØ showed smaller cell clusters joined by long projections which, as a typical IL-4 effect, are well documented and consistent with IL-4 treated mouse macrophages (Vereyken et al., 2011), possibly contributing to increased motility for migration to inflammation sites (Gordon, 2003).

Activation of poMoMØ led to significant changes in phenotype. Up-regulation of MHC and co-stimulatory molecules was consistent with the increased APC role of M1 activated macrophages (Gordon and Taylor, 2005). MHC-II up-regulation is a known effect of IFN-γ (Schroder et al., 2004), and CD86 expression alike was described in M1 macrophages (Mosser, 2003; Gordon and Taylor, 2005; Whyte et al., 2011). In addition CD25 was significantly increased on stimulated M1 poMoMØ, which as previously noted is a result of LPS in human monocytes (Scheibenbogen et al., 1992).

Important for migration (Geijtenbeek et al., 2000), DC-SIGN/CD209 expression is IL-4 dependent, associated with M2 macrophages (Martinez et al., 2006) and negatively regulated by IFNs (Relloso et al., 2002). Whilst we found that CD209 was significantly down-regulated in M1 MoMØ, we did not see IL-4 effects observed in other species. CD203a, significantly up-regulated on porcine M2 MoMØ, is a homolog to the human NPP1/CD203α, which regulates mineralization in articular cartilage and arterial tissues (Bollen et al., 2000; Goding et al., 2003), relating to the role of M2 macrophages in tissue repair.

Expression of both putative PRRSV-1 receptors CD163 and CD169 was unchanged in M1 or M2 MoMØ, which was particularly surprising in M2 MoMØ, given that others report this in human and mouse M2 macrophages, assumed to aid clearance of damaged cells (Gordon, 2003).

### Dexa and IL-10 Treatment of MoMØ Results in Two Further MoMØ Phenotypes

Dexamethasone and IL-10 induced morphological changes unlike those observed with IFN-γ/LPS and IL-4. The rounded appearance was comparable to a proposed ‘condensed’ morphology of dexa treated human macrophages (Porcheray et al., 2005), and consistent with the proposed deactivation state (Gordon, 2003). IL-10 induced only slight morphological changes, in agreement with Rey-Giraud et al. (2012) who described unchanged morphology of IL-10 treated human macrophages.

Both dexa and IL-10 significantly decreased CD83 expression on MoMØ, whilst only IL-10 decreased CD80/86. Decrease of maturation markers is in line with the assumption that both dexa and IL-10 are immunosuppressive. Whilst still poorly understood, deactivation is considered a final stage, halting inflammation and tissue damage (Gordon, 2003). Up-regulation of CD203a was the only shared effect of IL-10 and IL-4, not shown before, and not a feature attributed to alternative activation. IL-10 significantly increased endocytosis, thereby suggesting that uptake was independent of CD206 expression. Both dexa and IL-10 treatment significantly up-regulated CD163 in MoMØ, in line with the over-expression of CD163 described on differentiated macrophages treated with dexa and IL-10 (Porcheray et al., 2005).

Whilst earlier studies (Stein et al., 1992; Rey-Giraud et al., 2012) demonstrate increased endocytic and phagocytic activity in M2 macrophages, an increase was also observed in classically activated mouse macrophages (Vereyken et al., 2011). While neither M1 nor M2 treatment of poMoMØ significantly altered antigen uptake, dexa enhanced phagocytosis and IL-10 enhanced endocytosis. IL-10 has been shown to enhance endocytic activity of porcine macrophages (Montoya et al., 2009) and MoDC (Longoni et al., 1998). While early reports suggest that dexa suppresses macrophage phagocytic activity (Becker and Grasso, 1985), our result is in line with more recent studies using human MoMØ where dexa enhanced phagocytosis (Zahuczky et al., 2011).

### PRRSV-1 Susceptibility Varies across Different MoMØ Subsets

Previous studies have suggested that differences in disease susceptibility between pig breeds or individuals within pig breeds are correlated with differences in macrophage activation states (McCullough et al., 1993; Duan et al., 1997a; Ait-Ali et al., 2007).

Unchanged CD163 and CD169 expression following classical and alternative activation of MoMØ suggested that M1 or M2 MoMØ susceptibility to PRRSV-1 would be unaffected. We did not expect M1 macrophages to show such low replication levels. This failure, eventually overcome after 3 days, could be linked with the anti-viral effect of IFN-γ, which inhibits PRRSV-1 replication *in vitro* (Bautista and Molitor, 1999; Rowland et al., 2001).

Dexa significantly enhanced PRRSV-1 replication in MoMØ, associated with increased CD163 expression. Interestingly, dexa did not increase CD169, presumed the first receptor for PRRSV attachment (Calvert et al., 2007). Dexa’s ability to increase PRRSV replication with a significant effect on CD163 alone could suggest that CD169 was already sufficient before activation, or that it has no role in PRRSV, as suggested by Prather et al. (2013).

All MoMØ subsets appeared to increase their virus production into cell supernatant over time, however, the only significant differences observed between time-points were with IL-10 and dexa treatment up to 48 h p.i. Our data supports previous suggestions that one round of PRRSV replication in alveolar macrophages takes 9–16 h (Morilla, 2002), and suggests that further replication may have occurred subsequently.

Flow cytometric staining of PRRSV nucleocapsid protein was detected in dexa MoMØ only, which may be due to the low initial m.o.i. of 0.1. This was unexpected, since macrophages are considered the most favorable cell for PRRSV-1 tropism, where over time we expected a higher replication and spread through the culture. However, Thacker et al. (1998) detected only up to 38% of PRRSV-2 infected macrophages, using a m.o.i. of 1 (Thacker et al., 1998). It is thus reasonable to question if differentiated MØ are truly highly permissive for PRRSV.

### Porcine MoDC Show a Mature Phenotype in Response to Cytokine Activation Cocktail

Porcine MoDC differentiation has been described before (Paillot et al., 2001; Chamorro et al., 2004) and the morphology observed here fully aligned with previous reports and studies in other species (Miranda de Carvalho et al., 2006; Moyo et al., 2013). Similarly, porcine MoDC (poMoDC) maturation is well established and molecules involved in antigen presentation were up-regulated as described in response to LPS alone (Carrasco et al., 2001; Flores-Mendoza et al., 2008; Facci et al., 2010), or a LPS/IFN-γ/TNF-α cocktail (Pilon et al., 2009).

CD1a and CD1b are considered hallmark human DC phenotypes (Cao et al., 2002; Dascher and Brenner, 2003). Here, in pigs, CD1 expression was unchanged following maturation, which is consistent with a previous report (Carrasco et al., 2001). It should be noted, however, that CD1 diverged evolutionarily and appears in various isoforms differing between species. While the pig CD1 locus is described (Eguchi-Ogawa et al., 2007), we are unaware which CD1 molecule is detected by the antibody used.

Whilst CD83 is a known marker of DC maturation in both humans (Zhou and Tedder, 1996) and mice (Berchtold et al., 1999), recent studies highlight species differences regarding MoDC CD83 expression (Moyo et al., 2013). Although the expression was significantly up-regulated with maturation cocktail, CD83 expression in iMoDCs suggests that pigs are more similar to horses and unlike humans or mice which do not express CD83 on iMoDCs. CD206 expression is reportedly hallmark of human iDCs, absent on monocytes and mDC (Sallusto and Lanzavecchia, 1994; Mellman et al., 1998; Cochand et al., 1999), however, it would appear that poMoDCs are again different and rather similar to horses, where its modulation on MoDC is variable (Mauel et al., 2006).

CD14, a marker of monocytes and macrophages, not expressed by human blood-DCs (Thomas et al., 1993), was expressed by poMoDCs and decreased following maturation. CD14 is also expressed in MoDCs in other species including cat, cattle, and dog (Miranda de Carvalho et al., 2006). Some poMoDC studies (Paillot et al., 2001; Chamorro et al., 2004) suggest the absence of CD14 following maturation, although Carrasco et al. (2001) report increased CD14 following MoDC maturation (Carrasco et al., 2001). Such discrepancy could be owing to differences in the differentiation protocol. Carrasco et al. (2001) used autologous porcine serum for differentiation, often used for differentiation of macrophages, and these cells may have maintained some MoMØ characteristics.

### Porcine MoDC Respond to IL-10 and Dexamethasone by Modulation of Phenotype and Function

Whilst little was known about the effects of dexa on poMoDC prior to this study, reports suggested that glucocorticoids impair human MoDC differentiation and maturation (Woltman et al., 2000; Bengtsson et al., 2004), and that IL-10 induces a regulatory subset of DCs (Velten et al., 2004).

Dexa induced significant changes to MoDC phenotype, resulting in a phenotype unlike iMoDC nor mMoDC. MHC-II expression remained high, while the phenotype was otherwise inconsistent with maturation, also found to occur in human MoDCs treated with dexa (Duperrier et al., 2005). Differences between CD80/86 and CD83 positive cells in mMoDC and dexa MoDC support the theory that dexa inhibits maturation, as does our finding that dexa MoDCs express significantly increased CD14, exceeding that of porcine MoMØ. This finding aligns with other studies (Piemonti et al., 1999; Canning et al., 2000; Duperrier et al., 2005), and strengthens suggestion of an altered status. Importantly, CD163 and CD169 expression by poMoDC remained negligible following DC treatment with either dexa or IL-10. Unlike dexa, IL-10 failed to significantly modulate the MoDC phenotype, in line with IL-10 inhibition of human DC maturation (Buelens et al., 1997).

Despite variable endocytosis and phagocytosis, due to variability amongst animals, a significant difference was observed between the endocytic activity of dexa MoDC and iMoDC, and IL-10 increased phagocytic activity of poMoDCs, consistent with the increased antigen capture ability described in human IL-10-treated MoDCs, which maintain iMoDC characteristics (Morel et al., 1997). Increased endocytic activity was also described in humans (Longoni et al., 1998) and although not significant, our data confirm a trend to this effect.

### Infection of Porcine MoDC Subsets with PRRSV-1

Porcine reproductive and respiratory virus 1 replication in iMoDC remained low throughout the observation period of 72 h in both cells and cell supernatant. Another PRRSV-1 MoDC infection study also highlights variation between virus strains (Silva-Campa et al., 2010), suggesting that a strain specific feature of Lena might be to replicate poorly in MoDC. Interestingly, the same study suggests that DCs remain in an immature state following infection, as shown for PRRSV-2 strains (Wang et al., 2007; Flores-Mendoza et al., 2008), denoting that PRRSV favors replication in iMoDCs, consistent with data obtained here. It should be considered, however, that the maturation cocktail used contained IFN-γ, the anti-viral effects of which were discussed above.

Virus was not detected in supernatant until 24 h p.i., which mirrored the time-point where infection peaked in cells and indicated a significantly slower replication rate than in MoMØs. Interestingly, both dexa and IL-10 MoDCs reached peak virus replication even later. Absence of CD163 and CD169 on MoDCs, and the ability to infect such cells in principle, indicates that PRRSV infection of DCs may involve receptors not yet identified. It could be considered that DCs might become infected through uptake of bystander infected apoptotic cells. This, proposed by others (Frydas et al., 2013), would require further investigation using mixed cell cultures to demonstrate MoDCs infection as a consequence of other susceptible cells present. Whilst slow viral growth of PRRSV-2 has been associated with older pigs with increased viral resistance (Klinge et al., 2009), it is unlikely to explain the low infection levels observed in MoDCs here, since monocytes from the same animals showed high infection in parallel experiments.

In summary, PRRSV-1 is able to replicate in porcine myeloid cells in various states of activation or maturation, however, replication is much slower in MoDCs than in MoMØs. Dexa and IL-10 were both shown to promote PRRSV replication in macrophages, but failed to influence MoDCs in the same manner. Both IL-10 and dexa are known to act on MoDCs, but their inability to modulate CD163 and CD169 on these cells specifically makes up-regulation of these the most likely action by which PRRSV-1 replication is increased. Further investigation using a higher m.o.i. and measuring viral proteins in combination with CD163 and CD169 would be required to determine co-localization. Reports describing IL-10 production by PRRSV as a mechanism of immune suppression have been discussed controversially (Suradhat et al., 2003; Díaz et al., 2006). Our findings suggest an additional role of IL-10 in promoting PRRSV-1 replication. Given that DCs are considered the most professional APCs, the slow and inefficient infection of MoDCs may also explain the delay in T-lymphocyte response to PRRSV, possibly providing PRRSV with an elegant immune evasion mechanism.

## Author Contributions

FS, JP-F, and SG designed the study and all authors contributed to the acquisition, analysis and/or interpretation of data for further work. HS drafted the manuscript, which was further evaluated by all authors for its content and form. All authors have seen and finally approved the version submitted for publication.

## Conflict of Interest Statement

The authors declare that the research was conducted in the absence of any commercial or financial relationships that could be construed as a potential conflict of interest.

## Supplementary Material

**FIGURE S1 | Morphology of MoMØs.** Four-day-old MoMØs were either left unstimulated, **(A)**, or treated for 24 h with either IFN-γ and LPS to obtain M1-like MoMØs **(B)** or with IL-4 to obtain M2-like MoMØs **(C)**. Alternatively, four-day-old MoMØs were treated with either IL-10 **(D)**, or with dexa **(E)**. Light microscopy photographs were taken after 24 h with cytokines and are representative of three independent experiments. Magnification is 20×.

**FIGURE S2 | Data summarizing flow cytometric analysis of M1 and M2 MoMØ endocytosis.** The ability of unstimulated MoMØs or MoMØs treated with M1 or M2 cytokines to endocytose was assessed using APC*-labeled OVA. Cells were incubated with OVA at 4°C as a negative control (blue dots), or at 37°C (red dots), for 1 h before flow cytometric analysis of APC* fluorescence. Dots represent individual experiments, showing the percentage of cells fluorescing APC*, indicating endocytic OVA uptake. Lines represent mean percentage of cells associated with OVA-APC* ±SEM.

**FIGURE S3 | Data summarizing flow cytometric analysis of M1 and M2 MoMØ phagocytosis.** The ability of unstimulated MoMØs or MoMØs treated with M1 or M2 cytokines to phagocytose was assessed using FITC-labeled microsphere particles. Cells were incubated with particles at 4°C as a negative control (blue dots), or at 37°C (red dots), for 3 h before flow cytometric analysis of FITC fluorescence. Dots represent individual experiments, showing the percentage of cells fluorescing FITC, indicating phagocytic uptake. Lines represent mean percentage of cells associated with FITC microsphere particles ±SEM.

**FIGURE S4 | Endocytic Activity of MoMØs following dexa and IL-10 treatment.** The ability of unstimulated MoMØs or MoMØs treated with dexa or IL-10 to endocytose was assessed using APC*-labeled OVA. Cells were incubated with OVA for 1 h at 4°C (blue) or 37°C (red) before flow cytometric analysis of percentages of cells associated with APC*-OVA. Dots represent individual experiments; lines represent mean percentages ±SEM. One-way ANOVA was used to assess significance followed by Bonferroni’s multiple comparison test, **p* < 0.05.

**FIGURE S5 | Phagocytic Activity of Dexa and IL-10 MoMØs.** The ability of unstimulated MoMØs or MoMØs treated with dexa or IL-10 to phagocytose FITC-labeled microsphere particles was measured after 24 h of treatment with these factors. Cells were incubated with microsphere particles for 3 h, both at 4°C (blue) or 37°C (red) before flow cytometric analysis. Dots represent individual experiments, lines represent mean percentages ±SEM. One-way ANOVA was used to assess significance followed by Bonferroni’s multiple comparison test, **p* < 0.05.

**FIGURE S6 | Intracellular PRRSV staining using SDOW-17 in MoMØ subsets.** Monocytes were treated with M-CSF for 4 days to generate MoMØs, and either left unstimulated or activated with LPS and IFN-γ (M1), IL-4 (M2), dexa, or IL-10 for 24 h before infection with PRRSV Lena. At 20 h p.i cells were harvested and stained with a LIVE/DEAD Violet stain before being permeabilized and fixed for intracellular staining with α-PRRSV antibody SDOW-17 and secondary G1-APC* for detection by flow cytometry. Cells were gated on FSC/SSC **(A)** before dead cells were eliminated according to LIVE/DEAD Violet stain **(B)**. For negative controls, cells were stained with secondary G1-APC* only **(C)** and with a mouse IgG1 isotype and secondary G1-APC* **(D)**. SDOW-APC* staining is shown in mock infected MoMØs **(E)** and in unstimulated **(F)**, M1 **(G)**, M2 **(H)**, dexa **(I)**, IL-10 **(J)** treated MoMØs. Data are representative of three independent experiments.

**FIGURE S7 | Morphology of MoDCs.** Four-day-old MoDCs were either left untreated, **(A)**, or treated for 24 h with a maturation cocktail containing LPS, IFN-γ, IL-6, TNF-α, IL-1β, and PGE_2_ **(B)**. Alternatively, four-day-old MoDCs were treated for 24 h with IL-10 **(C)** or dexa **(D)**. Light microscopy photographs are representative of three independent experiments. Magnification is 20×.

## Abbreviations

DC: dendritic cell
dexa: dexamethasone
GM-CSF: granulocyte macrophage-colony stimulating factor
iDC: immature dendritic cell
LPS: lipopolysaccharide
M-CSF: macrophage-colony stimulating factor
mDC: mature dendritic cell
MØ: macrophage
MoDC: monocyte-derived dendritic cell
MoMØ: monocyte-derived macrophage
M1: MoMØ treated with IFN-γ/LPS
M2: MoMØ treated with IL-4
PBMC: peripheral blood mononuclear cell
pDC: plasmacytoid dendritic cell
PGE_2_: prostaglandin E_2_
PoMoDC: porcine monocyte-derived dendritic cell
PoMoMØ: porcine monocyte-derived macrophage
PRRSV: porcine reproductive and respiratory syndrome
