# Supplementary figures and images for: Establishing Porcine Monocyte-Derived Macrophage and Dendritic Cell Systems for Studying the Interaction with PRRSV-1
Source: Front Microbiol. 2016 Jun 2;7:832. doi: 10.3389/fmicb.2016.00832 (PMC4889594; doi:10.3389/fmicb.2016.00832)

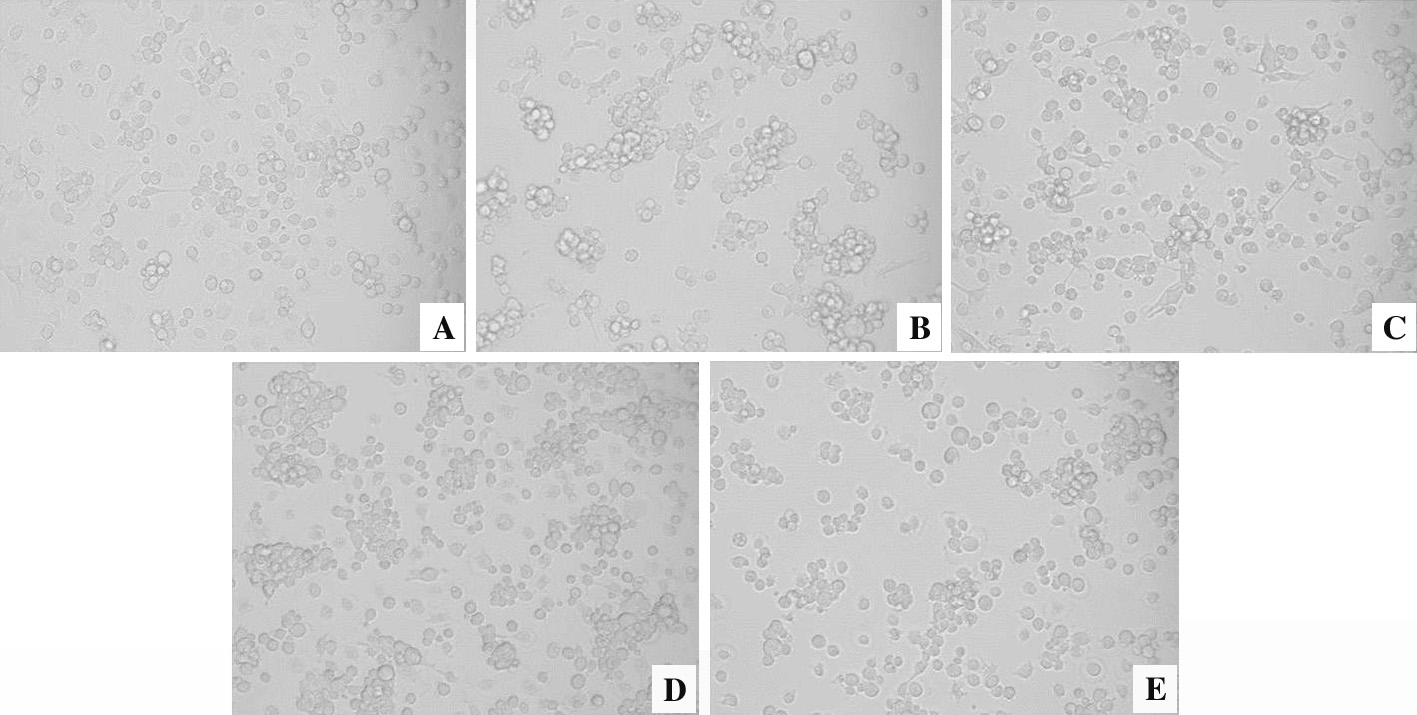

Supplement: Supplementary file 1 [file Image_1.JPEG]

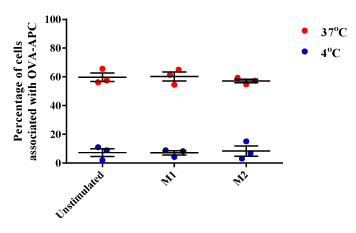

Supplement: Supplementary file 2 [file Image_2.JPEG]

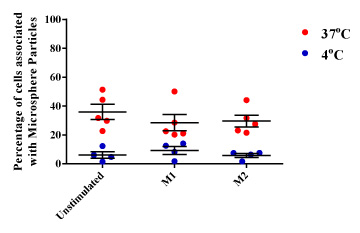

Supplement: Supplementary file 3 [file Image_3.JPEG]

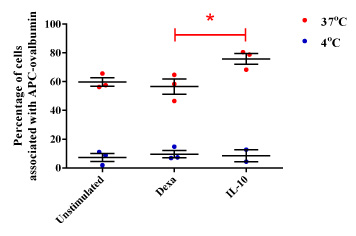

Supplement: Supplementary file 4 [file Image_4.JPEG]

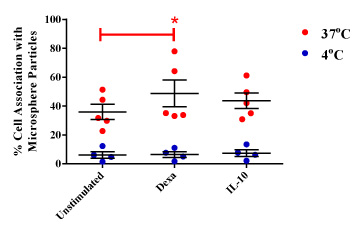

Supplement: Supplementary file 5 [file Image_5.JPEG]

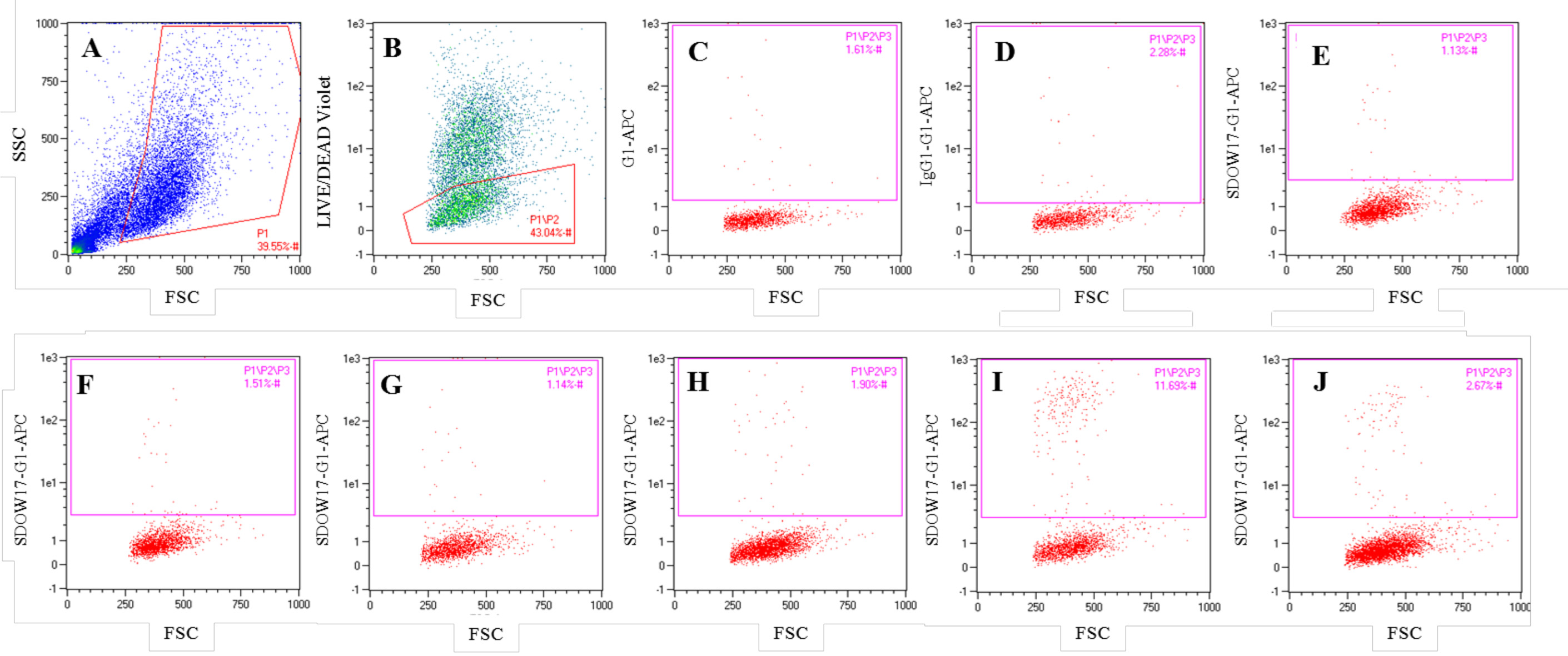

Supplement: Supplementary file 6 [file Image_6.JPEG]

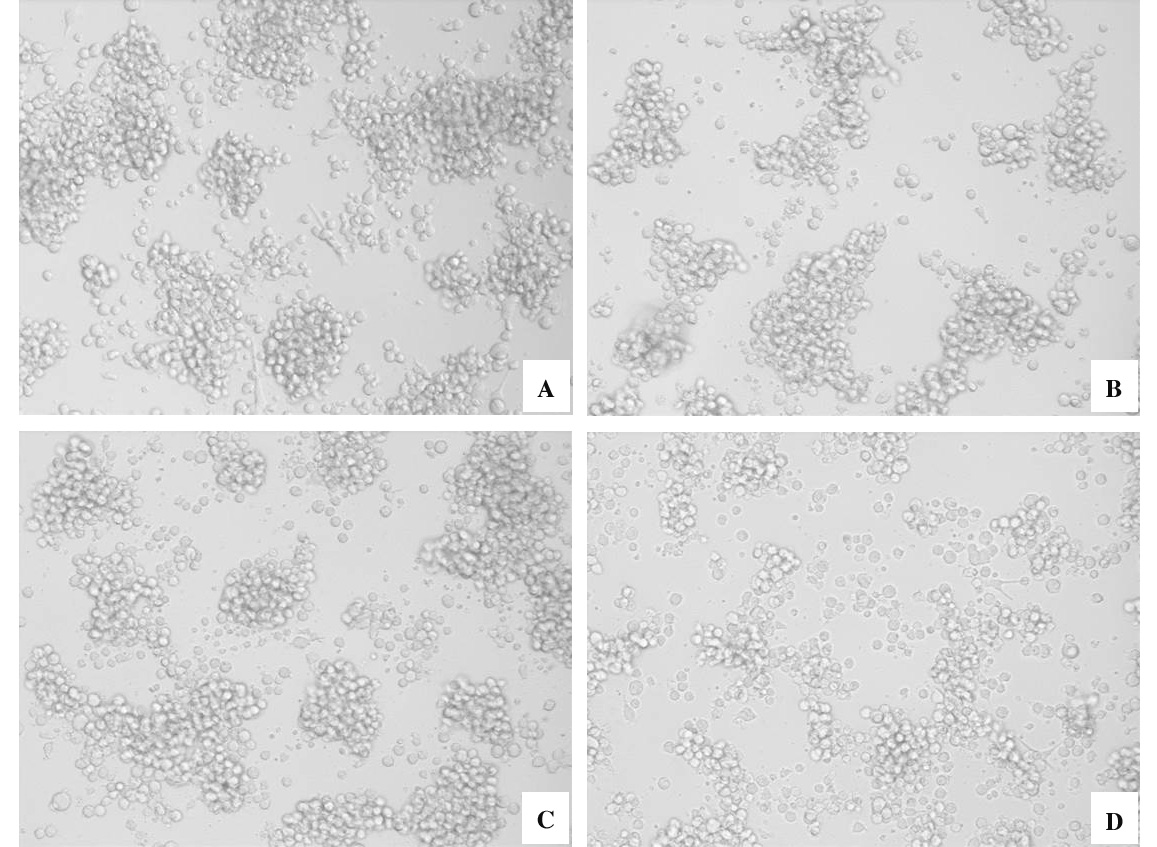

Supplement: Supplementary file 7 [file Image_7.JPEG]
